# Expression of the cytochrome P450s, *CYP6P3 *and *CYP6M2 *are significantly elevated in multiple pyrethroid resistant populations of *Anopheles gambiae s.s*. from Southern Benin and Nigeria

**DOI:** 10.1186/1471-2164-9-538

**Published:** 2008-11-13

**Authors:** Rousseau F Djouaka, Adekunle A Bakare, Ousmane N Coulibaly, Martin C Akogbeto, Hilary Ranson, Janet Hemingway, Clare Strode

**Affiliations:** 1 International Institute of Tropical Agriculture, Cotonou, 08BP0932, Benin; 2 Department of Zoology, University of Ibadan, Nigeria; 3 Centre de Recherche Entomologique de Cotonou, 06 BP 2604, Benin; 4 Vector Group, Liverpool School of Tropical Medicine, Pembroke Place, Liverpool, UK

## Abstract

**Background:**

Insecticide resistance in *Anopheles *mosquitoes is threatening the success of malaria control programmes. This is particularly true in Benin where pyrethroid resistance has been linked to the failure of insecticide treated bed nets. The role of mutations in the insecticide target sites in conferring resistance has been clearly established. In this study, the contribution of other potential resistance mechanisms was investigated in *Anopheles gambiae *s.s. from a number of localities in Southern Benin and Nigeria. The mosquitoes were sampled from a variety of breeding sites in a preliminary attempt to investigate the role of contamination of mosquito breeding sites in selecting for resistance in adult mosquitoes.

**Results:**

All mosquitoes sampled belonged to the M form of *An. gambiae *s.s. There were high levels of permethrin resistance in an agricultural area (Akron) and an urban area (Gbedjromede), low levels of resistance in mosquito samples from an oil contaminated site (Ojoo) and complete susceptibility in the rural Orogun location. The target site mutation *kdrW *was detected at high levels in two of the populations (Akron f = 0.86 and Gbedjromede f = 0.84) but was not detected in Ojoo or Orogun. Microarray analysis using the *Anopheles gambiae *detox chip identified two P450s, *CYP6P3 *and *CYP6M2 *up regulated in all three populations, the former was expressed at particularly high levels in the Akron (12.4-fold) and Ojoo (7.4-fold) populations compared to the susceptible population. Additional detoxification and redox genes were also over expressed in one or more populations including two cuticular pre-cursor genes which were elevated in two of the three resistant populations.

**Conclusion:**

Multiple resistance mechanisms incurred in the different breeding sites contribute to resistance to permethrin in Benin. The cytochrome P450 genes, *CYP6P3 *and *CYP6M2 *are upregulated in all three resistant populations analysed. Several additional potential resistance mechanisms were also identified that warrant further investigation. Metabolic genes were over expressed irrespective of the presence of *kdr*, the latter resistance mechanism being absent in one resistant population. The discovery that mosquitoes collected from different types of breeding sites display differing profiles of metabolic genes at the adult stage may reflect the influence of a range of xenobiotics on selecting for resistance in mosquitoes.

## Background

National Malaria Control Programmes are becoming increasingly reliant on strategies targeting the mosquito vectors. These almost invariably involve the use of long lasting insecticide treated nets (LLINs) or indoor residual spraying (IRS). Unfortunately the emergence of mosquito populations capable of withstanding insecticide exposure is threatening these control measures. In West Africa, *Anopheles gambiae *resistance to the four major classes of insecticides available for public health has been reported [1-6]. Pyrethroids are the only insecticide licensed for both LLINs and IRS, hence resistance to these insecticides is of concern, particularly as there has been a substantial increase (>60% coverage) in the number of people using bednets in Africa [7]. There are numerous reports of pyrethroid resistance throughout Africa [1,2,4,8,9] and a direct impact of this resistance on control programmes has been suggested in Mozambique and Benin [10,11].

Resistance can occur via target site insensitivity and/or metabolic detoxification. Target site resistance to pyrethroids and DDT in *An. gambiae *is due to a substitution at a single codon in the sodium channel gene, and is referred to as knock-down resistance (*kdr*). Two *kdr *alleles occur in *An. gambiae*, a leucine to phenylalanine substitution, known as West *kdr *[12] and a leucine to serine substitution known as East *kdr *[13]. N'Guessan [11] recently established a link between pyrethroid resistance caused by *kdr *and the failure of LLINs in Benin. Metabolic resistance is predominantly caused by elevated activity of one or more members of three large multigene enzyme families; cytochrome P450 monooxygenase (P450s), glutathione S-transferases (GSTs) and carboxylesterases (COEs). In *An. gambiae *these gene families have 111, 31 and 51 members respectively [14]. Recent work by Corbel *et. al*. using biochemical assays implicated the detoxification enzymes in conferring resistance to permethrin, DDT, dieldrin and carbosulfan in *An. gambiae *and *Culex quinquefasciatus *from four localities in Benin including rural, agricultural and urban sites [15].

Our understanding of metabolic resistance lags far behind that of pyrethroid target site resistance. Until recently this was due, in part, to a lack of sensitive tools with which to study resistance. Biochemical assays are useful in providing an indication of metabolic resistance, but only indicate whether there is a general P450, GST or COE response. With this in mind the *An. gambiae *detox chip was developed to identify specific members of these gene families up-regulated in association with insecticide resistance [16]. This tool has been used to screen several laboratory populations of *An. gambiae *[16-18] and a field population of *An. arabiensis *[19]. However this is the first report of the use of this tool to compare gene expression directly in neighbouring field populations that differ in their level of resistance.

The long term use of insecticides for controlling public health pests, beginning with the extensive use of DDT in the 1950's and 1960's, the more recent dramatic expansion in use of pyrethroid impregnated LLINs and extensive agricultural use of the same insecticides in and around mosquito breeding habitats, has led to widespread insecticide resistance in mosquito vector populations [20,21]. Agricultural use of pesticides is a particularly acute problem given that the same classes of insecticides are used in agriculture as public health but at comparatively elevated levels. An additional factor the environmental spillage of petroleum products has also been incriminated in selecting for resistance in malaria vectors in West Africa [22].

The type of responses and the mechanisms of resistance developed by mosquito populations when subjected to insecticide selection pressures have always been highly complex and difficult to predict. We hypothesized that the nature of the breeding sites could have a profound influence on the degree of resistance in the adult stage. Hence, in the current study, we deliberately selected mosquitoes from a variety of environments that were known to be contaminated with agricultural pesticides, petroleum products or urban pollutants and where there is an absence or in one case very limited vector control with insecticides. The aim of this study was to improve our knowledge of metabolic resistance in field populations of *An. gambiae *in Southern Benin and Nigeria.

## Results

### Genotyping and bioassay results

Individual mosquitoes from each locality were all identified as the M molecular form of *An. gambiae *s.s (Table 1.). East *kdr *detection was included in this study, as there is evidence that both mutations can occur in the same geographical location [23], but only West *kdr *[12] was found at high frequencies in both the Akron and Gbedjromede populations, and neither mutation occurred in Orogun or Ojoo. Following a one hour exposure to 0.75% permethrin, or in the case of Gbedjromede population a 10 min exposure to a permethrin impregnated net (Olyset^® ^impregnated nets at 1 g/m^2^) and subsequent 24 hour recovery period resistance was recorded in all 3 populations with mortalities of 23%, 36% and 80% for Akron, Gbedjromede and Ojoo respectively. The Orogun population was fully susceptible to permethrin. This is the first recorded evidence of pyrethroid resistance in the three localities.

**Table 1:** Molecular form, % mortality and *kdr *frequency of *An. gambiae *from Southern Benin and Nigeria

**Collection site**	**Cytotype (N = 30)**	**% Mortality (N = 80)**	**West *kdr *frequency (N = 30)**	**East *kdr *frequency (N = 30)**
Orogun (control site, peri-urban)	M	100*	0	0
Akron (agricultural)	M	23*	0.86	0
Ojoo (oil contamination)	M	80*	0	0
Gbedjromede (urban)	M	36†	0.83	0

*Mortality rate 24 hours post-exposure to 0.75% permethrin for 1 hour.

†Mortality rate following 10 min exposure to permethrin impregnated nets (Olyset impregnated nets at 1 g/m2)

### Comparison of gene expression in resistant and susceptible populations

The *An. gambiae *detox chip contains unique probes for each of the 193 genes from the three major detoxification gene families, plus probes for many of the genes associated with the oxidative stress response and other candidate genes associated with insecticide resistance from other studies. The detox array was used to compare gene expression in the susceptible Orogun population with each of the three resistant populations. RNA was extracted from pools of 10 mosquitoes that had survived permethrin exposure from each of the three resistance sites (Akron, Ojoo and Gbedjromede) and competitively hybridised with the susceptible Orogun population. Three independent replicates were prepared for each strain and dye swaps were performed for each pairwise comparison making a total of six arrays for each of the three populations analysed.

**Figure 1:**
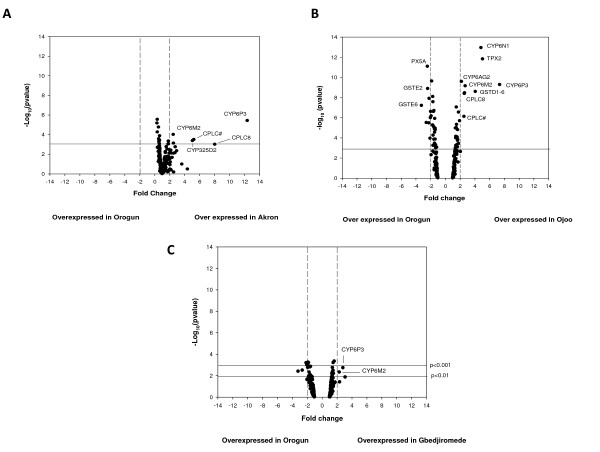
**Differential expression of *An. gambiae *detoxification genes in pyrethroid resistant and susceptible populations**. Pair-wise comparisons were made between the susceptible Orogun population and those from Akron (A), Ojoo (B) and Gbedjironmede(C). Differences are indicated as a function of both expression ratio (X-axis, vertical lines represent a 2 fold change) and significance expressed as the -log_10 _scale of the p-value of the t-test (y-axis, horizontal lines represent p < 0.001).

The results of the microarray experiments are summarised in Figure 1. Using a significance value of p < 0.001 and a fold change or ≥ 2, five genes had elevated expression in the resistant Akron population compared to the susceptible Orogun population. The most striking result was the 12.4-fold over expression of the cytochrome P450, *CYP6P3 *(figure 1A). Two additional P450s were also over expressed (*CYP6M2*, 2.5-fold and *CYP325D2*, 5.1-fold). Two cuticular precursor genes belonging to the low-sequence complexity group CPLC [24] were also over expressed by 5.2- and 3.4-fold.

The Ojoo site had lower levels of resistance, which is probably attributed to the absence of target site resistance in this population. When comparing gene expression in the survivors of insecticide exposure in this population with the susceptible Orogun population, nine genes were over-expressed. These included the two P450s elevated in the Akron site (CYP6P3: 7.4-fold and CYP6M2: 2.7-fold) and an additional two CYP6 P450s, *CYP6N1 *(4.8-fold) and *CYP6AG2 *(2.7-fold). (figure 1B). Two delta class GST genes were also over-expressed: *GSTD1-6 *(2.7-fold) and *GSTD11 *(2.1-fold). Both of the two cuticular pre-cursor genes, identified as over expressed in the Akron strain also showed elevated expression (2.6- and 2.5-fold respectively) and a peroxiredoxin, belonging to the typical 2-cysteine cytosolic group [25], TPX2 was over expressed 4.8-fold.

The Gbedjromede population, which was from an urban area also showed increased tolerance to permethrin in common with the Akron strain and yet the differences in gene expression, compared to the susceptible Orogun strain, were far less pronounced in this population (figure 1C). However, two of the P450s over expressed in the Akron and Ojoo sites were over expressed in the Gbedjromede site, although at a lower significance of p < 0.0019 for *CYP6P3 *(2.8-fold) and p < 0.0044 for *CYP6M2 *(2.4-fold). In contrast to the Akron and Ojoo populations neither of the cuticular pre-cursor genes showed differential expression.

## Discussion

The impact of pyrethroid resistance in *An. gambiae *from Southern Benin, believed to be driven by *kdr*, has been implicated in the failure of LLIN's in the region [11]. N'Guessan used biochemical analysis of the mosquitoes to identify metabolic resistance and ruled out the involvement of P450s, GSTs and COEs. However in a related study biochemical analysis of resistant populations of *An. gambiae *and *Culex quinquefasciatus *did indicate gross changes in all three enzyme families [15]. We have moved a stage further from biochemical analysis, by identifying metabolic genes up regulated in pyrethroid resistant strains. In a similar manner to Corbel *et al. *we analysed mosquitoes from breeding sites in urban and agricultural settings, but also included a breeding site where oil contamination is rife. To date studies involving the detox chip have been performed on mosquito populations which have often been subjected to long term laboratory colonisation and consequently may not mirror the genetic profile of field mosquitoes or they have compared field samples which are geographically unrelated, thereby potentially introducing variation. This is the first study to compare resistant field populations collected from the same geographical area (within 203 km), which have not been subjected to laboratory colonisation. Using this approach we identified four CYP6 and one CYP325 cytochrome P450s, two delta class GSTs, one peroxiredoxin and two cuticular precursor genes that are over-expressed in one or more resistant populations. Recent work within our group has demonstrated that selection of resistant populations of *An. gambiae *and *Aedes aegypti *with a number of insecticides (permethrin, deltamethrin and temephos) does not induce the expression of detoxification genes (Black, unpublished; Warr, unpublished). For this reason we believe that gene over-expression observed in this study is not due to transient up-regulation caused by the exposure of the adult mosquitoes to permethrin but instead reflects constitutive over expression of a distinct, but overlapping, subset of detoxification genes in each of these populations.

The gene showing the greatest levels of over-expression in all three populations is CYP6P3. *CYP6P3 *is the ortholog of *CYP6P9 *from *An. funestus*, a mosquito in which target-site resistance has not been reported. This gene has been genetically linked to pyrethroid resistance in *An. funestus *[26] and is highly over expressed in a pyrethroid resistant colony (FUMOZ-R) from Mozambique compared with the susceptible line FANG from Angola (>38 fold) [27]. Similarly in this study the *CYP6P3 *fold change between resistant and susceptible populations were the highest recorded by the detox chip for any gene to date (7.4-fold, Ojoo and 12.4-fold, Akron).

Interestingly Muller [19] employed the detox chip to investigate the effects on gene expression in *An. arabiensis *before and after a cotton spraying campaign and reported *CYP6P3 *to be down regulated post spraying. In that study the microarray comparison was made between field caught *An. arabiensis *from Cameroon and long-term laboratory reared *An. gambiae *Kisumu colony from Kenya thereby introducing variation caused by species differences, geographical location and lab colonisation.

*CYP6M2 *was also over-expressed in all three pyrethroid resistant populations. This gene is also up-regulated in pyrethroid resistant populations of *An. gambiae *from Ghana [28]. Microarray studies can only indicate associations between transcript levels and the phenotype of interest. However, recent studies on the *An. gambiae *recombinant proteins *CYP6P3 *and *CYP6M2 *have demonstrated that these enzymes are able to metabolise the pyrethroids permethrin, deltamethrin and cypermethrin *in vitro *(Stevenson, *personal commun*.). These combined results strongly support a role for both *CYP6M2 *and CYP6P3 in conferring pyrethroid resistance in *An. gambiae *from the localities sampled.

*GSTD1-6 *and *GSTD11 *belong to the delta class of GSTs with the former being one of four alternatively spliced variants of *GSTD1 *[29]. Whilst the delta class GSTs have been associated with resistance in other insects [30-32] this is the first study to implicate delta GSTs as conferring resistance in mosquitoes. GSTs and the peroxiredoxins are not thought to be able to metabolise pyrethroids directly. However both of these classes of enzymes can protect against oxidative stress and may counteract the pyrethroid induced oxidative stress encountered by the mosquitoes [25,31]. GSTs may also play a passive role in sequestering pyrethroids, thereby reducing the circulating levels of active insecticide [33].

The over expression of two cuticular precursor genes in both Akron and Ojoo resistant populations lends support to the hypothesis that mosquitoes may also protect themselves from insecticides by cuticular thickening, which leads to reduced penetration of insecticides. Compared with target-site and metabolic resistance, cuticular resistance is a less well understood mechanism and few studies have investigated the link between the insect cuticle and resistance [34-37]. However over expression of *CPLC8 *has very recently been demonstrated in pyrethroid resistant *An. gambiae *from Nigeria (Awolola, in press) and *An. stephensi *[38]. The *An. gambiae *detox chip only contains three of the estimated 295 putative cuticular proteins in this species [24], and all three of these belong to the CPLC family about which very little is known. The role of cuticular changes in resistance clearly warrants further investigation, as it may prove to be just as an important defence mechanism as metabolic and target-site resistance.

Little is known about the population structure of *An. gambiae *in Benin and no information is available on the level of gene flow between the different localities in this study. All the samples studied belonged to the M form of *An. gambiae*, which was expected as this is the main molecular form of *An. gambiae *in Southern Benin. Genetic introgression between the M and S forms does occur and is believed to be responsible the *ace-1*^*R *^mutation in a number of localities in Benin [39]. Further studies into metabolic resistance in the S form are needed before the role of introgression of these resistance mechanisms between different forms can be addressed.

The results observed do not correlate with geographic distance and instead neighboring populations, such as Ojoo and Orogun which are separated by just 7 km, show markedly different resistance phenotypes. Similarly despite a greater geographical distance from Orogun (>200 km) Akron and Gbedjromede demonstrated over expression of CYP6P3 and CYP6M in common with Ojoo mosquitoes. The current data suggest that the observed difference in expressions patterns may be due to the biological influence of the breeding sites. Individuals used in this study were collected as either 4^th ^instar larvae or pupae and therefore had been exposed to any contaminants in the breeding sites for the majority of their immature life stage. The three study sites were selected on the basis of the different indirect selection pressures that might be found in the mosquito breeding sites. The vegetable farm in Akron is heavily used and is permanently subjected to high doses of spraying of different families of agricultural pesticides against vegetable pests [40]. Residues of these sprayed pesticides have been detected in most *Anopheles *breeding sites located in areas of vegetable farming [20] and this probably leads to the selection of different mechanisms of resistance in field populations of *Anopheles*. The incrimination of agricultural pesticides in the selection of the *kdr *mutation has been documented in West Africa [1,6,20,41]. Akogbeto [20] further demonstrated that pyrethroid resistant *An. gambiae *larvae reared in water and soil samples taken from vegetable gardens in Benin were able to survive and proliferate in contrast to the susceptible phenotype.

Whilst the impact of agricultural use of insecticides has been widely linked to selection for resistance in malaria vectors, recent evidence has also implicated petroleum products [22,42]. Oil contamination in Ojoo occurs via spillage of petroleum products from mechanics, roadside petrol sellers and leakage from cars which are diluted by the rain and subsequently contaminate breeding pools. Petroleum products are also sometimes used as larvicides, and are attractive as they are relatively inexpensive and often easier to obtain than commercial insecticides. Petroleum products kill larvae not by suffocation but by contact toxicity and used at non-lethal doses may induce resistance. We can only speculate at this stage as to how oil contamination could potentially induce resistance. Hydrocarbons are the main component of petroleum products and structurally the aromatic hydrocarbons such as benzene and toluene show similarities with pyrethroids and could therefore potentially induce resistance over time

The third site under investigation was an urban setting. Rapid population growth leading to overcrowding, inferior housing and poor sanitation bring with it a different type of selection pressure on mosquito breeding sites caused by pollution from refuse and household waste. Other selection factors are the application of insecticides for vector control and the use of household insecticides in the form of sprays and coils. In Gbedjromede a malaria control programme in the form of the distribution of LLINs was recently implemented less than 3 months prior to larval collections. Previously non-insecticide impregnated bednets were used to deter mosquitoes. It seems unlikely that LLINs alone could account for the high level of pyrethroid resistance in this mosquito population in relatively short space of time, hence contamination of breeding sites, by as yet unidentified contaminants, could be a contributing factor.

Of particular significance is the fact that all resistant populations displayed evidence of metabolic resistance, irrespective of the presence of *kdr*, which was present at high frequency in the Akron and Gbedjromede populations, but absent from the Ojoo population. The *kdr *allele is believed to have spread to the M form from the S form via introgression [43]. The discovery of *kdr *in the M form echoes the findings of other studies [6,15,44]. It is difficult at this stage to determine the relative contributions of *kdr *and metabolic resistance in individuals with both mechanisms. It is however interesting to note that the highest level of resistance was observed in the Akron population, which has both *kdr *and metabolic resistance and the lowest resistance observed in the Ojoo population which displays only metabolic resistance. There was less evidence for metabolic resistance in the Gbedjromede population. However, these results should be interpreted with care as, due to problems with the availability of insecticide papers, the insecticide selection used to identify the most resistant subset of the population for microarray studies was less stringent than for the Akron and Ojoo population (i.e. 10 mins exposure to an Olyset^® ^impregnated net, compared with the a 60 minute exposure to 0.75% permethrin impregnated papers).

Given the evidence presented here, we postulate that xenobiotics prevailing in the different breeding sites have led to the over expression of a cohort of detoxification genes which in turn leads to cross resistance to insecticides. This study focused on resistance to pyrethroids due to their importance in malaria control but it would be interesting to investigate the influence of breeding sites on tolerance to alternative insecticide classes. Detailed HPLC-analysis of water and soil from the different breeding sites would be of great benefit in identifying the xenobiotics most likely to induce cross resistance to insecticides. The possibility that insecticide use outside malaria control activities in agricultural areas and xenobiotic contamination of breeding pools might be causing cross resistance to permethrin in *An. gambiae *has important implications for resistance management. For example, rotational use of different classes of insecticides in IRS programmes will be less effective if the selection pressure is coming from alternative sources. Hence further studies into the impact of the breeding site environment on resistance in adult mosquito populations are needed.

## Conclusion

In this study we have compared the gene expression profiles of resistant and susceptible populations of *An. gambiae *directly from the field without subjecting them to colonisation in the laboratory. We have produced evidence of metabolic resistance in mosquito samples from the Republic of Benin and Nigeria irrespective of the presence or absence of *kdr*. Furthermore we have identified two P450 genes, *CYP6P3 *and *CYP6M2 *which are strongly associated with permethrin resistance having been identified in all three resistant populations. In addition, preliminary evidence for a role for cuticular resistance is provided but this is an area that clearly needs further investigation.

## Methods

### Description of collection sites

**Figure 2:**
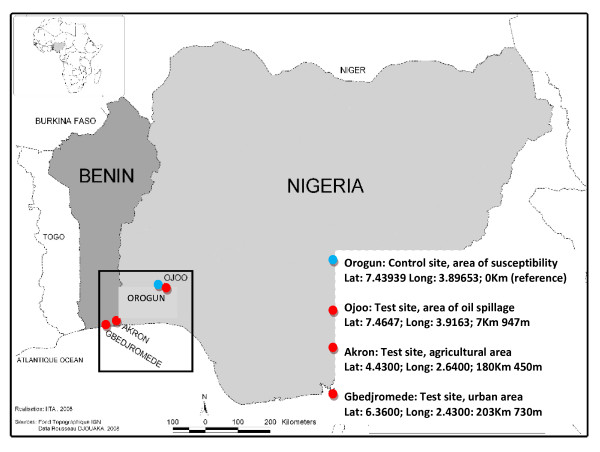
Map showing details of the four study sites and their locality in relation to the control site of Orogun.

The four field populations of *An. gambiae *used in this study originate either from South West Nigeria or South East Benin (Figure 2). *An. gambiae *samples were collected from three localities (Akron, Ojoo, Gbedjromede) where the presence of potential factors implicated in the selection of pyrethroid resistance had been previously reported. These factors include the massive use of agricultural pesticides by local farmers [40], the environmental spillage of petroleum products by mechanics and oil retailers [22] and urban pollution from household waste products. A control site, Orogun, where mosquito breeding sites were not under pyrethroid selection pressure, was located from ~7 – 200 km from the three test sites. Mosquitoes were collected from all sites during the dry season (December 2006–January 2007).

### The agricultural site of Akron

Located in South East Benin on the outskirt of Porto-Novo the capital city of the Republic of Benin, the agricultural site of Akron is the oldest vegetable farm, established by missionaries in 1945. The farm is now 20 hectares, employing about 150 workers typically growing cabbages, carrots, lettuce etc. Vegetable pests are targeted by massive use of pesticides mainly the carbamate Lannate^®^, pyrethroids (Decis^® ^and Fastac™) and the organophosphate malathion (Yandouleton, unpublished). The site is located in swampy areas providing an ideal environment for permanent breeding of mosquitoes.

### The oil spillage site of Ojoo

Ojoo is located in the south western part of Nigeria, in the state of Oyo. The spillage of petroleum products in the locality of Ojoo generates mainly from; spilled waste oil products from mechanics; oil leakages from old and poorly maintained cars; spilled oil from street retailers of petroleum products. Spilled on the ground, petroleum products are washed during rain falls and drain into *Anopheles *larval habitats where they aggregate into oily films clearly observable in most breeding sites recorded in this locality. There are no malaria control programmes (eg. ITNs, IRS or larviciding) in operation in Ojoo so people use electric fans to drive away mosquitoes.

### The urban site of Gbedjromede

The site of Gbedjromede is located in the urban perimeter of Cotonou, the economic capital of the Republic of Benin. This site is subjected to several urban organic pollutants which include waste water from households, house refuse, and industrial waste. The organic matter is swept during rain fall and drained into mosquito breeding sites. At Gbedjromede, *Anopheles *breeding sites are contaminated with urban pollutants which appear as decomposing organic particles in most surveyed larval habitats as evidenced by the naked eye. Four months prior to the collections at Gbedjromede the National Program of Malaria Control embarked on a large scale distribution of ITNs. Prior to this intervention non-impregnated ITNs were in use, and there is no history of IRS or larviciding.

### The locality of Orogun (control site)

Orogun is located in south western Nigeria in Oyo state. This site is at 10 Km from Ojoo and at approximately 150 km and 200 Km from Akron and Gbejromede respectively. Orogun is a rural area with neither the environmental spillage of petroleum products nor the use of pesticides by agricultural farmers. In this locality, *Anopheles *species are found breeding in clean water bodies. The insecticide susceptibility tests conducted with this population revealed the absence of resistance of this species to permethrin insecticide. Molecular analysis did not reveal any presence of the *kdr *target site mutation in individuals. Orogun was the closest site to the three test sites which still contained a susceptible population of *An. gambiae *and which share some basic molecular profiles with mosquitoes from the test populations. *An. gambiae *from Orogun were therefore considered the ideal field mosquito population which could be used during the sampling period as the control population for this study. Similar to Ojoo there is no history of ITN use, IRS or larviciding in Orogun.

### Mosquito collections

Fourth instar larvae and pupae were collected from the breeding pools and hence had individuals had spent a minimum of 5 days in contact with xenobiotics in their respective breeding sites. Larvae and pupae were maintained in the laboratory where the emerging F0 female adults were subsequently used for bioassays and molecular analysis.

### Genotyping of individuals

A single leg was removed from each individual mosquito from which gDNA was extracted using the Qiagen DNeasy Blood & Tissue Kit according to the manufacturer's instructions. The remaining body was used for RNA extraction and subsequent microarray analysis (see below). Species identification, molecular identification and East and West *kdr *detection were performed by PCR [12,13,45]. *Kdr *detection was performed on individuals who survived permethrin selection as described below.

### Selection of *Anopheles *populations with permethrin

The selection of resistant populations of *An. gambiae *"M" form used for microarray analysis in this study was based on WHO standard protocols for insecticide susceptibility testing using filter papers impregnated with diagnostic doses of permethrin (0.75%). A series of 20 females of *An. gambiae *(one day old) which had emerged from larvae collected directly from the field were introduced into WHO susceptibility kits containing papers coated with 0.75% permethrin. After an exposure time of one hour, mosquito samples from Akron and Ojoo were transferred into insecticide free tubes and maintained for 24 hours with sugar solutions. At the end of the 24 hr, dead mosquitoes were discarded and alive individuals (resistant population) were maintained until they reached three days old before being preserved in RNA-later for genotyping, *kdr *detection and microarray analysis. One-day old mosquitoes from Gbedjromede were selected by exposure to permethrin impregnated nets (Olyset^® ^impregnated nets at 1 g/m^2^) for 10 minutes and the survivors were then maintained on sugar solution until they reached 3 days-old. This method was adapted from the WHO cone bioassay for LLINs. The 3 minute recommended exposure was increased to 10 mins to increase the selection power.

The control population of Orogun were divided into two subsets. The first was used to establish the susceptibility level of the population and was subjected to a permethrin bioassay exactly as described for the Akron and Ojoo populations. The second subset was also subjected to the same bioassay but with control papers impregnated with silicon oil (insecticide carrier) instead of permethrin to control for any physiological stresses induced by the assay. It was this second subset of mosquitoes which were subsequently used as a control group for the microarray analysis (see below). A minimum of 30 females (3 pools of 10 females) of *An. gambiae *(three day old) were selected from the four populations.

### Target preparation and microarray hybridizations

RNA extractions, cDNA synthesis and labelling reactions were performed independently for each biological replicate. Total RNA was extracted from batches of 10 three-day old adult female mosquitoes using a PicoPure™ RNA isolation kit (Arcturus) according to manufacturer's instructions. Total RNA quantity and quality were assessed using Nanodrop spectrophotometer (Nanodrop Technologies, UK) before further use. RNA was amplified using a RiboAmp™ RNA amplification kit (Arcturus) according to the manufacturer's instructions. Amplified RNAs were checked for quantity and quality by spectrophotometry and agarose gel electrophoresis. Amplified RNA was reverse transcribed into labelled cDNA and hybridised to the array as previously described [17]. Each comparison was repeated three times with different biological samples. For each biological replicate, two hybridizations were performed in which the Cy3 and Cy5 labels were swapped between samples, hence a total of six hybridisations were performed for each comparison. Labelled cDNA from the Akron, Gbedjironmede and Ojoo sites were co-hybridised with the susceptible population from Orogun.

### Microarray Data Analysis

Spots that failed to meet any of the following criteria in either channel were rejected; (i) an intensity value of >300, (ii) signal-to-noise ratio of >3 and (iii) greater than 60% of pixel intensity superior to the median of the local background ± 2 SD. Normalisation and statistical analyses of the data was performed using the Limma 1.9 software package for R 2.3.1, available from the CRAN repository . Background corrected intensities from the red, (*R*, Cy5), and the green, (*G*, Cy3), channel were transformed to intensity log-ratios, *M *= logR/G, and their corresponding geometrical means, *A *= (logR + log G)/2. Within each array *M*-values were normalized as a function of *A *using the *Lowess *[46] scatter plot smoothing function and scaled to equalize the median absolute value across all arrays to account for technical biases between replicate hybridisations. Mean expression ratios were submitted to a one-sample Student's t-test against the baseline value of one (equal gene expression in both samples) with a multiple testing correction (Benjamini and Hochberg false discovery rate) [47]. Genes showing both t-test p values < 0.001 and ≥ 2-fold over or under expression were considered differentially expressed between comparisons. The expression data from these microarray experiments can be accessed at Vector base .

## Authors' contributions

RFD and CS conceived the study and participated in the implementation, data analysis, interpretation and manuscript preparation. AAB and JH guided the study from conception to the manuscript finalization. HR contributed in the study design and the write up of the manuscript. ONC and MCA participated in the design of the study. All authors read and approved the final manuscript.
